# The value of modular hemiarthroplasty for unstable femoral neck fractures in elderly patients with coxarthrosis

**DOI:** 10.1186/s12891-016-1068-x

**Published:** 2016-05-23

**Authors:** Robert Döring, Thorsten Jentzsch, Max J. Scheyerer, William Pfäffli, Clément M. L. Werner

**Affiliations:** Department of Trauma Surgery, University Hospital Zürich, Rämistrasse 100, 8091, Zürich, Switzerland

**Keywords:** Femoral neck fracture, Bipolar hemiarthroplasty, Osteoarthritis and coxarthrosis, Double fond osteophyte (DFO), Posterior wall sign (PWS)

## Abstract

**Background:**

Displaced femoral neck fractures are common in the elderly patient. The surgical treatment options consist of a hemiarthroplasty (HA) or total hip arthroplasty (THA). However, the best surgical choice is still under debate. Bipolar HAs do not address preexisting arthritic changes of the acetabulum, which may lead to an unfavorable clinical outcome. The purpose of the present study was to conduct a long term follow-up analysis of the bipolar hemiarthroplasty with particular focus on the influence of preoperative acetabular osteoarthritis on the functional outcome.

**Methods:**

In a retrospective observational study, the medical charts of consecutive patients treated with a bipolar hemiarthroplasty at a level one trauma center between 2004 and 2008 were reviewed before a final radiographic and clinical follow-up was performed. The outcome variables consisted of arthritic findings on the pre- and postoperative x-rays with particular focus on double fond osteophyte (DFO) and posterior wall sign (PWS) as well as the revision rate and functional scores.

**Results:**

This study included 102 patients with a mean age of 77.2 years. Most patients (75 %) had a Kellgren-Lawrence grading scale (KLGS) of 2 or 3. While only 30 % of patients had a DFO, most patients (73 %) had a PWS. The DFO correlated significantly with the KLGS, but no correlation was seen with the clinical outcome. Most patients showed a decreased offset by a mean of −7.8 mm. The mean modified Harris Hip Score (HHS) of 90.3 and the mean Merle d'Aubigné score of 10.8 correlated significantly. Despite a significant correlation of the HSS subcategory of pain and the preoperative KLGS, there was no statistical relationship between the arthritic x-ray measurements and the clinical outcome.

**Conclusions:**

In the presented study population, the presence of radiographic acetabular osteoarthritis did not influence the clinical outcome after bipolar hemiarthroplasty for displaced femoral neck fractures.

## Background

Displaced femoral neck fractures are common injuries of the elderly patient. In western countries, they are treated in nearly every trauma center. Respecting the entities of an aging population and an increasing contribution of developing countries, the estimated worldwide incidence of hip fractures in 2050 is over six million cases per year [1]. Also considering rising insurance costs, femoral neck fractures represent an important public health problem. It is well known that the most frequently affected patient collective of patients older than 60 years is subject to an increased disability, morbidity, and mortality [2, 3]. Therefore, an appropriate fracture treatment is obligatory.

If an operative therapy is indicated, there are two widely accepted options, i.e. monopolar or bipolar hemiarthroplasty (HA) and total hip arthroplasty (THA); both with optional use of bone cement. However, the optimal treatment with the best clinical outcome is still under debate [4]. While some authors prefer bipolar HA due to the advantages of smaller dislocation rates, less complex surgery, shorter surgical time, and lower initial costs, recent studies point toward a slightly better function and patient satisfaction with THA; especially in healthy elderly patients with good mental conditions [4–13]. This might be explained by different factors. Hemiarthroplasties in general do not address preexisting arthritic changes of the acetabulum, which may lead to restriction of movement and hip pain. Further acetabular erosion might occur, potentially resulting in early revision and conversion to THA. Therefore, some authors name osteoarthritis as a contraindication for HA [14], but a recent study showed no correlation between the grade of preoperative osteoarthritis and functional outcome scores [15].

In case of bipolar HA, it has been shown that the cup rotates to a relatively constant horizontal position after weight bearing, which is attributed to the overall effect of different factors such as joint geometry, muscle action, soft tissue, and metal-on-bone friction [4, 16, 17]. Even though the importance of this balanced position for the clinical outcome remains unclear, it might be possible that arthritic changes of the acetabulum, with central osteophytes in particular, result in an aberrance of the head position and lead to restricted range of motion or hip pain [18]. Despite the advantage of modular components to reconstruct a wide spectrum of anatomical characteristics and in the management of recurrent dislocations of hip hemiarthroplasty [19, 20], there are still hardware-related disadvantages. One important biomechanical feature is the inaccuracy of the femoral offset, which has been shown to play an important role in the range of motion [21–26] and in functional outcome after hemiarthroplasty [27].

The aim of the present study was to conduct a long term follow-up analysis of bipolar hemiarthroplasty. A particular focus was placed on the preoperative osteoarthritis of the acetabulum with special attention on the influence of the double fond osteophyte (DFO), the posterior wall sign (PWS), and the femoral offset on the functional outcome.

## Methods

The study was approved by the local ethics committee (Cantonal Ethic Committee of Zurich, KEK-ZH-Nr. 2011–0320) and written consent was obtained from all patients. All patients with femoral neck fractures, who were treated operatively at a level one trauma center between 2004 and 2008 were retrospectively evaluated. Patients were identified by reviewing medical and surgical records. The time period was chosen to compromise between a long follow-up period, an acceptable number of survivors and the availability of x-rays. A further inclusion criterion was the implantation of a bipolar hemiarthroplasty (Mathys AG, Bettlach, Switzerland). Exclusion criteria were age younger than 18 years, pathological fractures secondary to malignant disease and polytrauma patients.

**Fig. 1:**
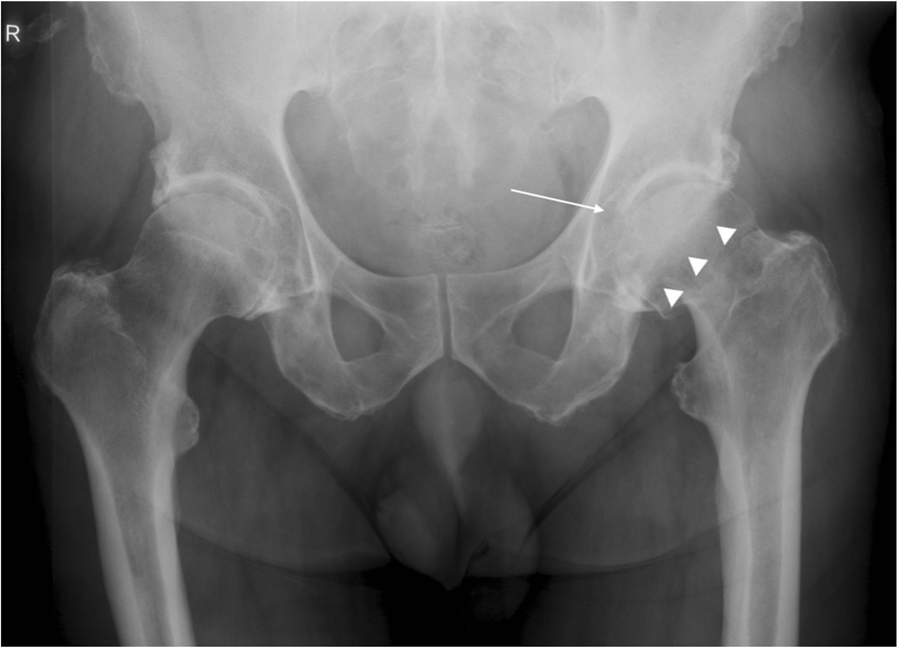
Pelvic X-ray with signs of arthrosis consisting of a double fond osteophyte (DFO) (*thin arrow on the left*) and a posterior wall sign (PWS) (*thick arrows on the right*)

In a first step, the preoperative x-rays were evaluated for several variables consisting of typical signs of osteoarthritis, i.e. joint space narrowing, subchondral sclerosis, osteophytes, which particularly included the DFO, cysts as well as the PWS (Fig. 1) [28]. The grade of osteoarthritis was determined using the Kellgren-Lawrence grading scale (KLGS) [29]. Any revisions of the affected hip were retrieved from the medical charts and patients were asked for revisions during clinical follow-up. In the case of missing data or death, the primary care physician was contacted by phone. Correlations between preoperative osteoarthritis and revision rate with THA were calculated.

In a second step, patients were invited to a follow-up examination in the outpatient clinic. In this step, cases with revision of the arthroplasty or conversion to monopolar HA or THA were excluded in order to compare clinical outcome measures. Patients received a questionnaire composed of the scientifically and clinically validated short form (SF)-36-Score, which included the sub-groups of physical functioning (PF), role physical (RP), bodily pain (P), general health perception (GHP), role emotional (RE), social functioning (SF), mental health (MH), and vitality (EF), as well as the Merle-d’Aubigé-Score (MAS), and the modified Harris Hip Score (HHS) [30–32]. A clinical examination of the hip joint was performed in order to determine any localized pain and to assess the range of motion.

In a third step, two standardized x-rays of the pelvis were obtained through an anterior-posterior projection with 20° internal rotation of the legs and an axial cross-table projection. In cases where a follow-up examination was not possible, the last archived x-ray was used. Then, the operated side was compared to the contralateral side with regard to the femoral offset and leg length. Femoral offset was determined by measuring the distance between the center of rotation of the femoral head and the proximal long axis of the femur. In case of arthroplasty the distance between the center of rotation of the femoral head and a line bisecting the long axis of the stem was measured. To identify leg length discrepancies, a reference line connecting the upper margin of both acetabula was drawn. In the following, the perpendicular distance between this line and each minor trochanter was measured and compared.

**Fig. 2:**
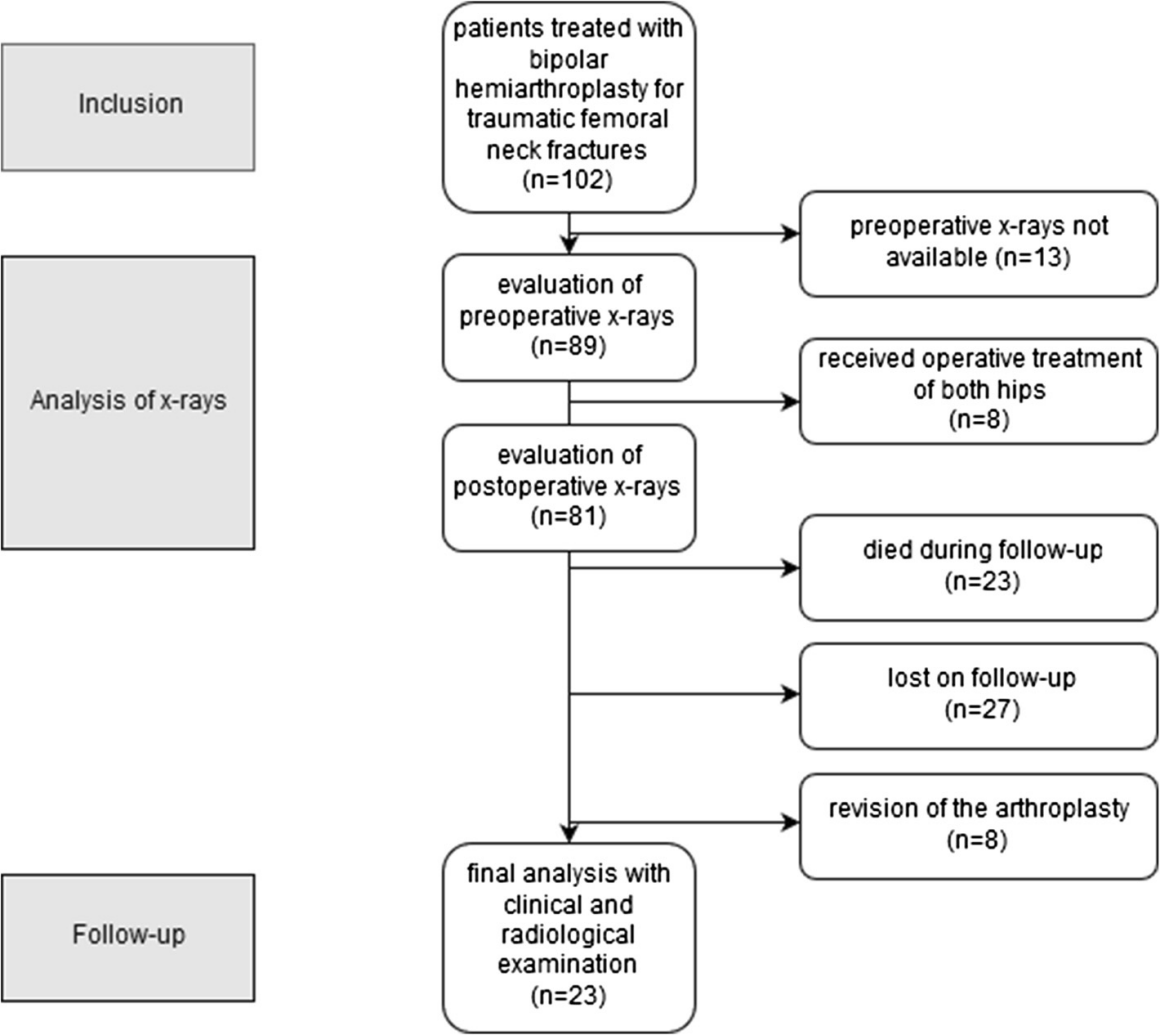
Flow Chart of the study

All data were entered into Excel (Microsoft Corp., Redmond, USA) and exported to the Statistical Package for the Social Sciences (SPSS) (version 21.0; IBM Corp., Chicago, IL, USA) for statistical analysis. Mean values and their standard deviation are given. Differences between particular groups were analyzed using Pearson’s chi-squared test or Mann-Whitney U test and 95 % confidence intervals are given. The Spearman’s rank correlation coefficient was used for the comparison of different continuous measures of arthritis and different clinical scores, while the phi coefficient measured the association for binary measures of arthritis. A value of *p* < 0.05 was considered statistically significant. All statistics were supervised by a local statistician (Fig. 2).

## Results

**Fig. 3:**
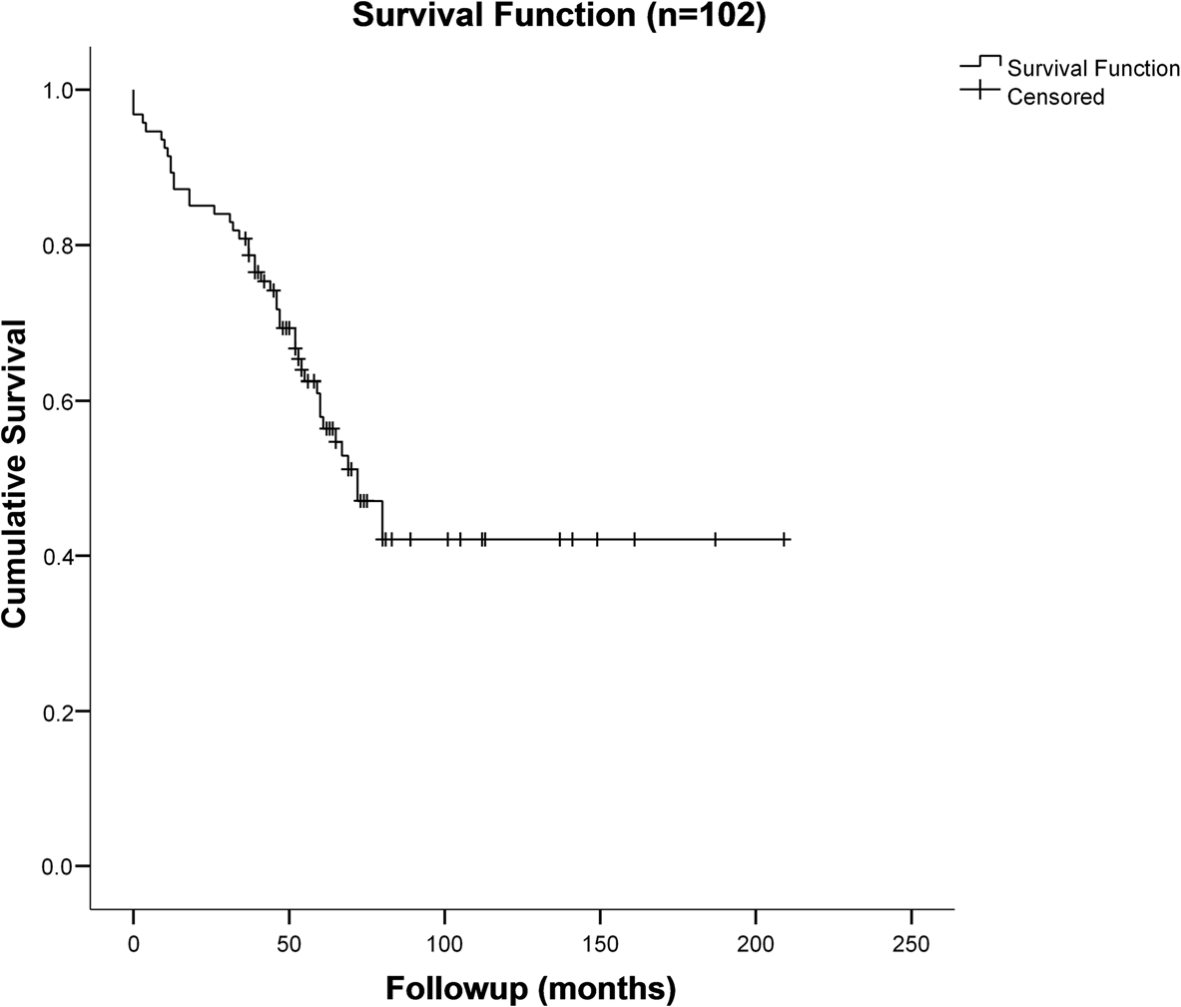
Kaplan-Meier survival curve showing the survival of patients with the cumulative (cum) survival on the *y axis* and the time to death in months on the *x axis*. Mean for survival time 113.2 months (95 % CI 93.4, 133.0)

One hundred and two patients were enrolled in this study. The mean age at the day of the operative treatment was 76.9 (range 43 to 96) years. Seventy-five (74 %) patients were older than 70 years. The sex ratio was 1.0:2.5 (29 (28 %) males to 73 (72 %) females) (Table 1). A total of 44 (43 %) patients had died during the follow up period (Fig. 3). The anatomical side of the fracture was evenly distributed with 54 (53 %) fractures on the left and 48 (47 %) fractures on the right side. Eighty four (82 %) patients received a cemented and 18 (18 %) an uncemented hemiarthroplasty. In 13 (13 %) cases, a revision was necessary, whereof nine (9 %) patients received a total hip replacement.

**Table 1:** Patient characteristics (*n* = 102)

	*n* (%)
Gender	
Male	29 (28)
Female	73 (72)
Age (mean) (standard deviation (SD))	76.9 (SD 11)
Side of fracture	
Left	54 (53)
Right	48 (47)
Cemented hemiarthroplasty	84 (82)
Reoperation	13 (13)
Reoperation with total hip replacement	9 (9)

### Analysis of preoperative X-rays

**Fig. 4:**
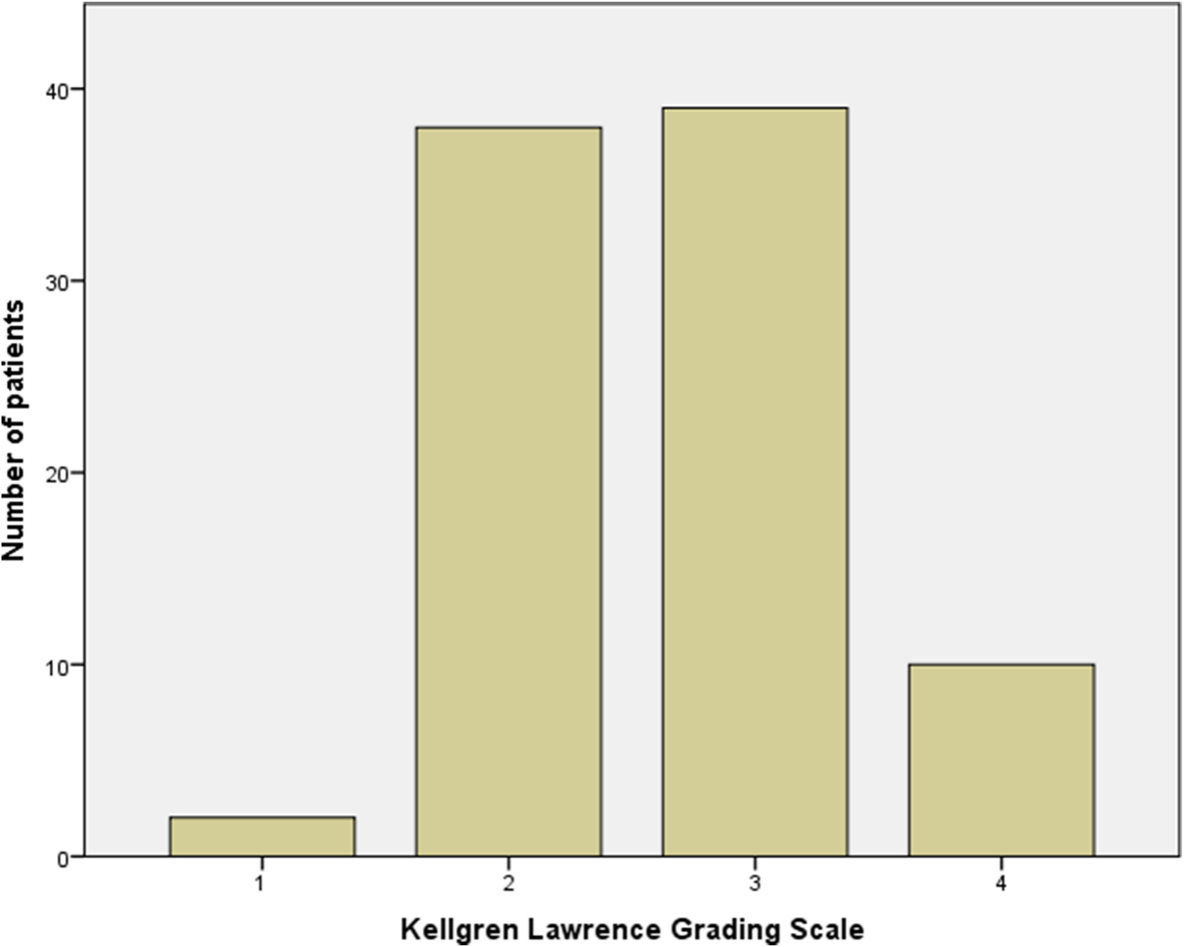
Grade of osteoarthritis according to the Kellgren Lawrence grading scale (KLGS) in preoperative x-rays (*n* = 89)

Preoperative x-rays of 89 (87 %) patients could be evaluated. Most (37 and 38 %, respectively) patients showed a grade of 2 or 3 on the KLGS for osteoarthritis (Fig. 4, Table 2). Double fond osteophytes were present in 31 (30 %) cases; while a positive PWS was found in 74 (73 %) cases. A positive correlation was observed between the existence of DFOs and the KLGS (*p* < 0.05). There was no correlation between the existence of DFO (phi coefficient 0.15, *p* = 0.55) or the grade of osteoarthritis (Spearman’s rho 0.05, *p* = 0.1) and revisions with total hip replacement.

**Table 2:** Preoperative x-rays (*n* = 89, missing 13)

	*n* (%)
Gender	
Male	25 (28)
Female	64 (72)
Age (mean) (standard deviation (SD))	77.2 (SD 11.2)
Grade of osteoarthritis according to KLGS	
Grade 1	2 (2)
Grade 2	38 (43)
Grade 3	39 (44)
Grade 4	10 (11)
Double fond osteophyte (DFO)	31 (35)
Posterior wall sign (PWS)	74 (83)

### Analysis of postoperative X-rays

**Fig. 5:**
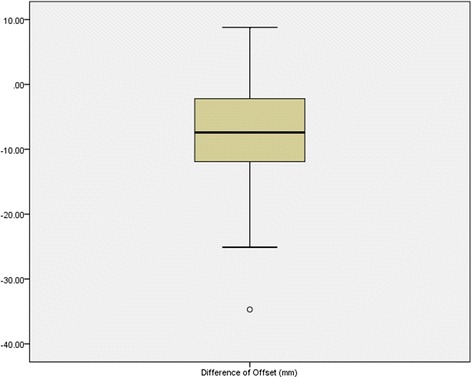
Box plot illustrating the difference between the femoral offset of the operated hip to the opposite side in postoperative x-rays (*n* = 81)

Eighty-one (79 %) patients (25 males and 56 females, mean age 76.6 (SD 11.2)) were available for the evaluation of a postoperative x-ray since the non-affected hip had also received operative treatment rendering a comparison unfeasible in 8 cases (Table 3). The mean femoral offset after bipolar hemiarthroplasty was 39.6 (SD 8.5, range 15–58) millimeters (mm). In the majority (*n* = 70 (69 %)) of the cases, a decreased offset was noted on the operated side when compared to the contralateral side (Fig. 5). The mean difference of offset was −7.8 (SD 7.9, range −35 to7) mm. The radiologically measured leg length discrepancy was 2.3 (SD 8.0) mm.

**Table 3:** Follow-up functional data (*n* = 23, missing 79)

	*n* (%)
Gender	
Male	4 (17)
Female	19 (83)
Age (mean) (standard deviation (SD))	70.2 (SD 8.2)
Hip pain [n]	10 (43)
Range of motion [°] (mean) (standard deviation (SD))	
Flexion	99.6 (SD 9.6)
Internal rotation	27.6 (SD 10.4)
External rotation	39.3 (SD 6.6)
Abduction	42.1 (SD 6.9)
Adduction	19.1 (SD 2.4)
Harris Hip score	90.3 (SD 15.3)
Pain	40.2 (SD 8.7)
Function	41.3 (SD 8.9)
Merle d’Aubigné score	10.8 (SD 2.3)
SF-36 score (mean) (standard deviation (SD))	
Physical functioning (PF)	42.7 (SD 19.6)
Role physical (RP)	59.1 (SD 21.9)
Bodily pain (BP)	57.7 (SD 24.5)
General health (GH)	50.8 (SD 14.8)
Vitality (VT)	49.4 (SD 17.7)
Social functioning (SF)	52.6 (SD 12.8)
Role emotional (RE)	55.5 (SD 12.3)
Mental health (MH)	52.5 (SD 13.8)

### Follow-up

Clinical data of 23 (23 %) patients were available for the final analysis. The mean time from the implantation of the bipolar prosthesis to the follow-up examination was 93.9 (SD 45.6) months. The mean modified HHS was 90.3 (SD 15.3) points and the mean Merle d'Aubigné score was 10.8 (SD 2.3) points. There was a statistically significant correlation between both scores (*p* < 0.001). The points for the SF-36 are shown in Table 3. Furthermore, there was a positive correlation between the subcategory of HHS pain and the preoperative grade of osteoarthritis (*p* = 0.02), but no other statistical relationship was found for the remaining scores or clinical measures and preoperative x-ray findings (Table 3 and 4).

**Table 4:** Influence of osteoarthritis on functional outcome (*p*-values, *n* = 23, missing 79; *p < 0.05)

Patients functional outcome	KLGS	DFO
Flexion	0.683	0.957
Internal rotation	0.978	0.829
External rotation	0.157	0.649
Abduction	0.309	0.579
Adduction	0.303	0.593
Harris Hip score	0.289	0.235
Pain	0.015*	0.610
Function	0.652	0.379
Merle d'Aubigné score	0.053	0.397
SF-36 score		
Physical functioning (PF)	0.897	0.137
Role physical (RP)	0.173	0.302
Bodily pain (BP)	0.971	0.957
General health (GH)	0.357	0.393
Vitality (VT)	0.812	0.063
Social functioning (SF)	0.942	0.028
Role emotional (RE)	0.924	0.305
Mental health (MH)	0.608	0.710

## Discussion

Femoral neck fractures of the elderly represent an important health care problem. It is widely accepted that an arthroplasty is mandatory for these fractures, but the correct choice of either HA or THA may still be debated in certain cases [4–9]. In contrast to THA, modular HAs are usually implanted in the elderly with less functional demands because they are associated with shorter surgical duration and less blood loss, which leads to lower revision rates. A combined finite element and clinical study by Ichihashi et al. suggested cautious use of bipolar HAs in cases of osteoarthritis due to the migration of the outer head of bipolar HAs [18]. Besides, studies by Sah and Estok as well as Carulli at al. have pointed out the usefulness of dual mobility cup prostheses in cases of dislocations of HAs [19, 20]. The purpose of the present study was to investigate the influence of preexisting acetabular arthritic changes on the clinical outcome after bipolar hemiarthroplasty for displaced femoral neck fractures. The presented results indicate no differences between the radiographic presence of osteoarthritis according to the KLGS, the presence of a DFO or a PWS and the clinical outcome.

To determine the femoral offset, standardized x-rays of the pelvis in anterior-posterior projection with 20° internal rotation of the legs were used. Here, the measured value can easily be underestimated and influenced by hip rotation [33]. A solution was published by Lechler et al., who developed a method of assessing rotation-corrected femoral offsets [34, 35]. However, this method was only used for proximal femoral nailing and is not yet validated for HA. Therefore, and to achieve comparability to a larger number of other studies, the projected femoral offset was used in consideration of the accuracy of x-rays. When comparing the femoral offset on the operated and healthy sides, the mean femoral offset was −7.8 mm indicating smaller femoral offsets on the side of the HA. Previous studies have shown that a higher femoral offset leads to a better lever arm for the abductors, which ultimately increases the range of motion [23–26]. This was particularly illustrated in a study by McGrory et al., who showed a significant correlation between the femoral offset and the abductor lever arm, range of motion as well as strength [23]. Furthermore, the restoration of the original femoral offset has been shown to be associated with smaller risks of dislocation as well as less strain and decreased wear of the prosthesis in a thorough review by Lecerf et al. [22]. In a recent study, Buecking et al. analyzed the influence of femoral offset on functional outcome in bipolar HA. A mean femoral offset of 36.9 mm was found, which is comparable to the present study (39.6 mm) [27]. However, the positive correlation between femoral offset and HHS could not be reproduced.

The proportion of revisions (13 %) was similar to previous studies, which showed substantially higher proportions for HAs than THAs [3, 13]. In 9 cases a conversion to THA was performed. Due to the long follow-up period of more than seven years and the advanced age of the study population, definite reasons for these procedures could not be evaluated. Nevertheless, possible explanations could include pain, lack of motion or dislocation, which might be related to preoperative osteoarthritis. Still, no correlation could be found. Furthermore, the mortality rate of 44 % is in line with a previous study of van den Bekerom, who studied 307 bipolar HAs and reported a mortality rate of 28 % after one and 63 % after five years [3].

The presented overall clinical results according to the HHS and Merle d’Aubigné are good [30, 31]. The average scores of 90.3 in the HSS surpass those of previous studies [10, 12]. For example, Bezwada et al. reported a mean HHS of 82 points after bipolar HA in 248 patients [10]. While 56 % of patients were also pain-free, studies by Bezwada et al. as well as Casserly and Healy stated values around 60 and 69 %, respectively [10, 11]. A potential explanation for this lower number of pain-free patients may be attributed to the advanced age, which is usually associated with more comorbidities and longer follow up periods.

There are several limitations to this study. Although a final follow-up clinical visit was scheduled, several patients had been lost in the realm of this retrospective study. This led to rather small subgroups and impeded a meaningful statistical analysis at times. The radiographic femoral offset measurements may have been confounded by rotational differences in our standardized x-rays. Although a goniometer was used for the clinical investigation, values had to be rounded to intervals of five degrees, possible leading to measurement errors. Inter-rater discrepancies were also not stratified for. Furthermore, several comorbidities of this elderly cohort complicated data retrieval at times. Therefore, prospective trials may consider these aspects in their evaluation of the topics presented herein. An example of a future study would consist of a comparison between HA and THA in a large sample size with respect to influence of preoperative arthritic changes on the outcome.

## Conclusion

In the presented study population, the presence of radiographic osteoarthritis did not influence the clinical outcome after bipolar hemiarthroplasty for displaced femoral neck fractures.

### Ethics approval and consent to participate

The study was approved by Cantonal Ethic Committee of Zurich, KEK-ZH-Nr. 2011–0320. Written consent was obtained from all participating patients.

### Availability of data and materials

All relevant data supporting the conclusions of this article are included within the article/tables.

## Abbreviations

DFO: double fond osteophyte
HA: hemiarthroplasty
HHS: Harris hip score
KLGS: Kellgren-Lawrence grading scale
MAS: Merle-d’Aubigné-score
PWS: posterior wall sign
THA: total hip arthroplasty
